# Challenges in institutional ethical review process and approval for international multicenter clinical studies in lower and middle-income countries: the case of PARITY study

**DOI:** 10.3389/fped.2024.1460377

**Published:** 2024-11-05

**Authors:** Eliana Lopez-Baron, Qalab Abbas, Paula Caporal, Asya Agulnik, Jonah E. Attebery, Adrian Holloway, Niranjan “Tex” Kissoon, Celia Isabel Mulgado-Aguas, Kokou Amegan-Aho, Marianne Majdalani, Carmen Ocampo, Havugarurema Pascal, Erika Miller, Aimable Kanyamuhunga, Atnafu Mekonnen Tekleab, Tigist Bacha, Sebastian González-Dambrauskas, Adnan T. Bhutta, Teresa B. Kortz, Srinivas Murthy, Kenneth E. Remy, the Global Health Subgroup of the Pediatric Acute Lung Injury and Sepsis Investigators (PALISI) Network

**Affiliations:** ^1^ Departamento de Pediatría y Cuidado Crítico Pediátrico, Hospital Pablo Tobón Uribe, Universidad EIA, Medellín, Colombia; ^2^ Departamento de Pediatría, Universidad de Antioquia, Medellín, Colombia; ^3^ Department of Pediatrics and Child Health, Aga Khan University Hospital, Karachi, Pakistan; ^4^ Health Systems Program, Department of International Health, Johns Hopkins Bloomberg School of Public Health, Baltimore, MD, United States; ^5^ Red Colaborativa Pediátrica de Latinoamérica (LARed Network), La Plata, Argentina; ^6^ Division of Critical Care and Pulmonary Medicine, Department of Pediatrics, St Jude Children’s Research Hospital, Memphis, TN, United States; ^7^ Department of Global Pediatric Medicine, St Jude Children’s Research Hospital, Memphis, TN, United States; ^8^ Department of Neurosurgery, Barrow Global, Barrow Neurological Institute, St. Joseph’s Hospital and Medical Center, Phoenix, AZ, United States; ^9^ Division of Pediatric Critical Care Medicine, Department of Pediatrics, University of Maryland School of Medicine, Baltimore, MD, United States; ^10^ Department of Pediatrics, University of British Columbia, Vancouver, BC, Canada; ^11^ Division of Critical Care, British Columbia Children’s Hospital, Vancouver, BC, Canada; ^12^ Unidad de Terapia Intensiva Pediátrica, Hospital General León, León, México; ^13^ Department of Pediatrics and Child Health, School of Medicine, University of Health and Allied Sciences, Ho, Ghana; ^14^ Division of Pediatric Intensive Care Unit, Department of Pediatrics and Adolescent Medicine, American University of Beirut Medical Center, Beirut, Lebanon; ^15^ Grupo Quirón Salud, Clínica Imbanaco, Cali, Colombia; ^16^ Department of Pediatrics and Child Health, University Teaching Hospital of Butare, Butare, Rwanda; ^17^ Department of Pediatrics and Child Health, University Teaching Hospital of Kigali, Kigali, Rwanda; ^18^ Department of Pediatrics and Child Health, St. Paul’s Hospital Millennium Hospital Medical College, Addis Ababa, Ethiopia; ^19^ Red Colaborativa Pediátrica de Latinoamérica (LARed Network), Montevideo, Uruguay; ^20^ Departamento de Pediatría y Unidad de Cuidados Intensivos de Niños del Centro Hospitalario Pereira Rossell, Facultad de Medicina, Universidad de la República, Montevideo, Uruguay; ^21^ Division of Pediatric Critical Care Medicine, Department of Pediatrics, Indiana University School of Medicine, Indianapolis, IA, United States; ^22^ Division of Critical Care, Department of Pediatrics, University of California, San Francisco, CA, United States; ^23^ Institute for Global Health Sciences, UCSF, San Francisco, CA, United States; ^24^ Department of Anesthesiology, Pharmacology and Therapeutics, University of British Columbia, Vancouver, BC, Canada; ^25^ Division of Pediatric Critical Care Medicine, Department of Pediatrics, Rainbow Babies and Children’s Hospital, Case Western Reserve University, Cleveland, OH, United States

**Keywords:** ethics, low- and middle-income countries, research, global, challenges, Institutional Review Boards, IRBs

## Abstract

**Background:**

One of the greatest challenges to conducting multicenter research studies in low and middle-income countries (LMICs) is the heterogeneity in regulatory processes across sites. Previous studies have reported variations in requirements with a lack of standardization in the Institutional Review Board (IRB) processes between centers, imposing barriers for approval, participation, and development of multicenter research.

**Objectives:**

To describe the regulatory process, variability and challenges faced by pediatric researchers in LMICs during the IRB process of an international multicenter observational point prevalence study (Global PARITY).

**Design:**

A 16-question multiple-choice online survey was sent to site principal investigators (PIs) at PARITY study participating centers to explore characteristics of the IRB process, costs, and barriers to research approval. A shorter survey was employed for sites that expressed interest in participating in Global PARITY and started the approval process, but ultimately did not participate in data collection (non-participating sites) to assess IRB characteristics.

**Results:**

Of the 91 sites that sought IRB approval, 46 were successful in obtaining approval and finishing the data collection process. The survey was completed by 46 (100%) participating centers and 21 (47%) non-participating centers. There was a significant difference between participating and non-participating sites in IRB approval of a waiver consent and in the requirement for a legal review of the protocol. The greatest challenge to research identified by non-participating sites was a lack of research time and the lack of institutional support.

**Conclusions:**

Global collaborative research is crucial to increase our understanding of pediatric critical care conditions in hospitals of all resource-levels and IRBs are required to ensure that this research complies with ethical standards. Critical barriers restrict research activities in some resource limiting countries. Increasing the efficiency and accessibility of local IRB review could greatly impact participation of resource limited sites and enrollment of vulnerable populations.

## 1 Introduction

Despite advancements in treatments, the burden of pediatric critical care diseases in low- and middle-income countries (LMICs) remains significant, with notable disparities in survival rates compared to high-income countries (HICs), hence amplifying the need for worldwide multicenter collaborative research. The research procedure requires formal institutional approval with the involvement of Institutional Review Boards (IRBs), which are responsible for reviewing the study protocol to ensure compliance with ethical research standards (1, 2). Nonetheless, due to the lack of standardization in this process, significant variability in IRB functioning has been previously evidenced in terms of revisions, time to protocol approval, and consent requirements, among other factors, which hinders the involvement of centers from LMICs that could benefit from these studies in assessing conditions that, if improved, could enhance outcomes for children with critical illnesses (3, 4).

The ethical approval process poses a significant challenge for researchers from LMICs when conducting international studies, in addition to the barriers encountered prior to IRB submission, as they typically possess less experience in submitting studies for IRB review and have limited support for the associated administrative processes. Michelson et al. have established in an observational pediatric multicenter study that this variability in regulatory monitoring is a time-consuming process that affects study participation, ultimately leading to the withdrawal of international researchers from trials (5). Furthermore, the COVID-19 pandemic exacerbated the heterogeneity and complexity of site IRBs, with certain boards introducing stronger regulations while others imposed less strict ones (6). The burden of IRB issues faced by researchers from low- and middle-income countries is still unknown.

The objective of the study was to elucidate the IRB related barriers to participation in multinational studies conducted in LMICs, and to investigate the challenges associated with the submission process and execution of research that may have limited site participation during the Global PARITY study (Pediatric Acute cRitical Illness sTudY), a prospective, observational, multicenter, multinational point prevalence study designed for assessing the burden of acute pediatric critical illness in LMICs (7).

## 2 Methods

### 2.1 Study design, setting and population

We used the Global PARITY study platform to evaluate the regulatory processes at each participating site and sites that expressed interest in participating in Global PARITY and started the approval process, but ultimately did not participate in data collection or non-participating sites. Global PARITY was an unfunded prospective, observational, multicenter, multinational point prevalence study conducted in 46 resource-limited hospitals across North, Central, and South America, Africa, the Middle East; and South Asia. Global PARITY measured the prevalence of pediatric acute critical illness, associated outcomes and resource utilization at four time points throughout one year (July 2021–July 2022). One of the pre-planned secondary studies was the present survey exploring the IRB hurdles encountered during the research process. Participating research sites for this study were recruited via established relationships among physician-led pediatric critical care research networks including the World Federation of Pediatric Intensive & Critical Care Societies (WFPICCS), the Global Health subgroup of the Pediatric Acute Lung Injury and Sepsis Investigators (PALISI) Network (www.palisiglobalhealth.org), Red Colaborativa Pediátrica de Latinoamérica (LARed Network). Global PARITY was coordinated by the Department of Pediatrics at the University of Maryland and has been deemed exempt by the University of Maryland (IRB, HP-00086107). Participating sites were required to obtain local Institutional Review Board (IRB) approval prior to participation.

### 2.3 Survey development and distribution

A subgroup of Global PARITY investigators and core coordinators with expertise in global pediatric critical care research in low- and middle-income countries created 16 multiple-choice and categorical questions survey. The survey inquired about the frequency of IRB meetings, committee composition, submission and reply timeframes, and IRB requirements for language translation, costs, and data sharing agreements. The survey was administered in English and Spanish, based on the locations of the sites, and was evaluated by researchers prior to distribution to the lead investigators at each site. We intended to restrict the survey to a completion time of under 15 min. The survey's introduction page contained a statement outlining the goal of the questionnaire, its duration, and the researchers conducting the study. A consent statement was incorporated in the survey's introduction. Following the construction of the survey, the Principal Investigator (PI) from each of the 46 participating sites was requested to complete the ethical approval survey after the initial two sampling periods, with a reminder provided one week later. The survey additionally encompassed demographic data regarding the participant locations.

An additional shorter survey version was created for sites who indicated interest in participating in Global PARITY and began with the approval process but ultimately did not participate (non-participating sites) in order to examine their IRB characteristics. This survey included questions developed for the participating sites survey, as well as nine Likert-type questions categorizing barriers to research development into five levels (not a barrier; somewhat a barrier; neutral; moderate barrier; significant barrier), based on how they perceived those barriers would interfere with carrying out research projects. Barriers studied were institutional support, time, financial support, staff availability, ethical approval, and information access. The University of Maryland's Research Electronic Data Capture (REDCap) application was utilized to collect survey responses (8).

### 2.4 Statistical analysis

The characteristics of the institutions were analyzed according to the nature of the variables. Categorical variables were analyzed using absolute and relative frequencies. Quantitative variables were described, depending on their distribution, using means or medians and their respective measures of dispersion (standard deviation, interquartile range or percentiles). Comparison between participant and non-participant sites were performed using the *t*-test, Fisheŕs exact test, or Chi-square test depending on the type and distribution of the variable. Results of the Likert-type questions were analyzed as an ordinal variable using medians and percentiles, and relative frequencies were displayed on a bar graph. Analysis was done using R version 4.2.1.

## 3 Results

A total of 91 sites pursued local IRB approval. Forty-six sites (50%) were approved and accepted to collect data. The survey was electronically sent to the principal investigators of all centers, with response rates of 46/46 (100%) from participating centers and 21/45 (47%) from non-participant centers for a total of 67 sites. The role of principal investigators was physician in 57% (*N* = 38) of the surveyed centers, and 75% (*N* = 51) of the institutions were classified by survey respondents as public hospitals with university affiliation. Table 1 shows the characteristics of the institutions and role of the principal investigator in both participating and non-participant institutions.

**Table 1:** Characteristics of participant and non-participant institutions.

	Total (%) *n* = 67	Participants (%) *n* = 46	Non-participants (%) *n* = 21
Role of the principal investigator			
Assistant physician	38 (57)	22 (48)	16 (76)
Administrator	8 (12)	3 (7)	5 (24)
Clinical or medical officer	8 (12)	8 (17)	0
Medical intern	6 (9)	6 (13)	0
Resident	6 (9)	6 (13)	0
Medical student	1 (1)	1 (2)	0
Type of hospital			
Public	50 (75)	35 (77)	15 (71)
Private	14 (21)	9 (19)	5 (24)
Both	2 (3)	1 (2)	1 (5)
Don’t know	1 (1)	1 (2)	0
University affiliation			
Yes	52 (78)	33 (72)	19 (90)
No	15 (22)	13 (28)	2 (10)

### 3.1 Characteristics of IRB process

Table 2 depicts the characteristics of IRB process for both participant and non-participant centers. IRB committees met once every one to two months at 56% (*N* = 37) of sites. The average time needed for a typical research protocol approval was 35 days (range 19.5–71 days) for participating sites and 32 days (range 15.2–57.8 days) for non-participant sites. There was no difference in the associated cost for the IRB process or mandatory translation into the local language between participant and non-participant sites. Only two non-participant institutions disclosed the values of the costs of the IRB process, so no numerical cost comparisons could be done.

**Table 2:** Characteristics of the ethics committee/IRB process.

Characteristics of IRB process	Total (%)	Participants (%)	Non participants (%)	*P* value	No data*
Days for ethics approval [*n* = 45, Me (IQR)]	35 (17–66)	35 (19.5–71)	32 (15.2–57.8)		22
Frequency of ethics committee review (*n* = 66)				0.016	1
Weekly	14 (21)	10 (22)	4 (20)		
Every 1–2 months	37 (56)	24 (52)	13 (65)		
Every 3–6 months	6 (9)	6 (13)	0		
>6 months	9 (14)	6 (13)	3 (15)		
Costs associated with IRB (*n* = 66)				0.2	1
Yes	14 (21)	12 (27)	2 (10)		
No	52 (79)	33 (73)	19 (90)		
Translation into local language mandatory (*n* = 67)				0.4	
Yes	31 (46)	23 (50)	8 (38)		
No	36 (54)	23 (50)	13 (62)		
Professional translation requirement (*n* = 29)				0.4	2
Yes	7 (24)	4 (19)	3 (37.5)		
No	22 (76)	17 (81)	5 (62.5)		

* Sites without data provided.

### 3.2 IRB characteristics

There was no significant difference between participant and non-participant sites in terms of full IRB review requirements for study protocol approval, but there was a significant difference between whether the IRB authorized a waiver of consent (82% vs. 47%, respectively, *p* = 0.015). There was also a significant difference in the requirement for a legal review of the protocol; participating sites required a legal review less frequently than non-participating sites (24% vs. 52%, respectively, *p* = 0.021). Other IRB requirements did not vary significantly between participating and non-participating sites (Table 3).

**Table 3:** IRB characteristics for all the sites.

IRB functioning	Total (%)	Participants (%)	Non participants (%)	*P* value	No data^a^
Full board review to grant approval (*n* = 62)				0.5	5
Yes	47 (76)	36 (78)	11 (69)		
No	15 (24)	10 (22)	5 (31)		
IRB approval of waived consent (*n* = 60)				0.015	7
Yes	44 (73)	37 (82)	7 (47)		
No	16 (27)	8 (18)	8 (53)		
Data Sharing agreement (DTA) requirement (*n* = 65)				0.2	2
Yes	15 (23)	8 (18)	7 (33)		
No	50 (77)	36 (82)	14 (67)		
Approval from a separate institution, without local review (*n* = 67)				0.7	
Yes	12 (18)	9 (19)	3 (14)		
No	55 (82)	37 (81)	18 (86)		
Legal review of protocol (*n* = 67)				0.021	
Yes	22 (33)	11 (24)	11 (52)		
No	45 (67)	35 (76)	10 (48)		

^a^ Different sample sizes are found for some variables due to missing data.

### 3.3 Characteristics of the IRB in non-participant sites

Table 4 provides a detailed analysis of the characteristics, barriers, and opportunities to participate in research for non-participating sites. Seventy-one percent of the non-participating sites (*N* = 15) reported having previously participated in multicenter research studies; however 95% (*N* = 19) reported not having protected time, 71% (*N* = 15) not receiving institutional support for research. Furthermore, 63% (*N* = 14) indicated a lack of research-trained investigators supporting the research process (epidemiologist, statistics). Sixty-two percent (*n* = 13) of those questioned about the advantages of undertaking research for academic or institutional purposes did not mention any advantages.

**Table 4:** Characteristics of the research process and barriers to research in non-participant sites.

Characteristics of research process	Non participants (%) *n* = 21
Benefit/advantage of doing research^a^	
Economic	2 (10)
Time for research	2 (10)
Academic	9 (43)
None	13 (62)
Protected time to research^b^	
Yes	1 (5)
No	19 (95)
Availability of support for research (epidemiologist, writing manuscripts, translation)	
Yes	6 (29)
No	15 (71)
Previous participation in research	
Yes	15 (71)
No	6 (29)
Previous participation in multicenter multinational research	
Yes	13 (62)
No	8 (38)
If so, did this result in a publication?	
Yes	14 (88)
No	1 (12)
Additional research training	
Yes	7 (33)
No	14 (63)
Perceived Barriers^a^	Median (P25–75)
Limited time	4 (3–5)
Lack of institutional support	3 (2–5)
Difficulty on data extraction	3 (2–4)
Availability of staff for data collection	3 (2–5)
Costs of doing research	2 (2–4)
IRB approval	3 (2–4)
Lack of familiarity with research	3 (2–3)
Legal barriers	2 (2–3)
Access to internet or technology	1 (1–2)

^a^ More than one option could be selected.

^b^ One institution did not report data.

**Figure 1:**
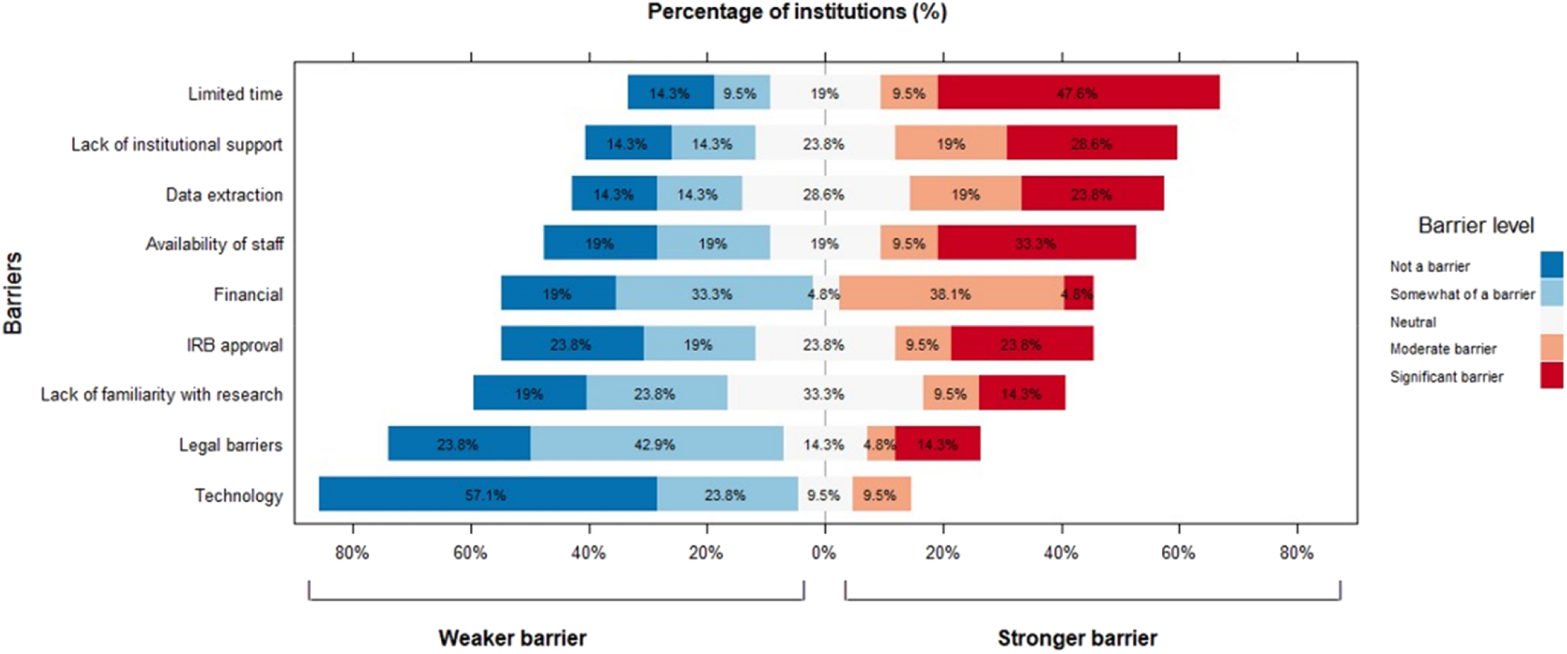
Barriers for research participation among non-participant institutions.

Among the barriers, the lack of time received the highest score (Median [P25-P75]: 4 [2-4]), followed by the availability of staff to support data collection, lack of institutional support, IRB approval process, difficulty in data extraction, and lack of familiarity with research. Legal restrictions and access to technology for research collaboration were not seen as significant barriers. The results of this survey are also depicted in Figure 1 as relative frequencies. The lack of time was identified as a significant barrier by almost 50% of the institutions, while more than 20% considered the lack of institutional support, difficulty in data extraction, and availability of staff to support data collection as significant barriers.

## 4 Discussion

This study allowed us to assess the barriers encountered during IRB approval for a multinational collaborative research study, the Global PARITY, by centers in LMICs, as well as the key aspects of the IRB process for obtaining research approval. It is important to emphasize that of all the centers intended to participate, only 50% ultimately did, underscoring the significance of this study in assessing the challenges to participation. This represents an essential initial step toward addressing these barriers and enhancing the involvement of resource-limited centers in future studies, with the objective of improving outcomes in such settings and reducing disparities.

Despite receiving approval from the study's lead center and being classified as a minimal risk study, most of the hospitals involved in this study required their own full board review, demonstrating not only a lack of standardization but also a lack of centralized IRB approval, which can delay data collection or prevent some centers from participating. These findings are consistent with an earlier international research project reported by Michelson et al, the sepsis prevalence, outcomes, and therapies study (SPROUT) study, which demonstrated a high degree of variability in the methods of review and protocol approval despite a previous lead center approval, which impacted participation (5). Resistance to using a centralized IRB for multisite trials comes from concerns about the importance of local context and the lack of committees that follow international guidelines, as well as uncertainty about the quality of other IRBs and the impact of research on the local community, as demonstrated by Levine et al. for researching in a global emergency setting (9). International guidelines applicable in high-resource settings have addressed the utilization of a central Institutional Review Board (IRB) for multisite studies to mitigate delays and the potential introduction of bias. This has been recommended for research regulatory organizations, and several multicenter studies in high-resource countries have been successfully conducted through centralization and standardization (10, 11). This would be a crucial factor to take into account for LMICs in established regional groups, as it will help standardize international guidelines that take into account local considerations and maintain ethical research principles.

We have also found that non-participating sites face barriers that prevent them from participating in the study. This is a remarkable finding because prior studies have not gone into great detail about this issue, underscoring the significance of our findings. The primary barriers to participation in PARITY included the lack of protected research time, insufficient research resources, a deficit in the research workforce and training, and a significant absence of institutional support, which hinders centers engagement in all phases of the research process, from conceptualization to data collection and analysis. An additional barrier that was found was the lack of formal training in research, which caused a delay in the process and avoided a more in-depth local data analysis. The issues outlined are comparable to those identified by Levine et al. in their evaluation of the IRB process for research in pediatric emergency settings (9). It is widely acknowledged that augmenting the workforce for research elevates institutional costs, a crucial factor particularly when resources are constrained. However, the lack of research personnel, designated time for research, or adequately trained staff results in an increased workload, leading to a decline in research activity and the associated advantages of engaging in research. The involvement of center directors and their awareness of the significance of research as a preliminary measure in enhancing outcomes could optimize workload distribution and facilitate the incorporation of research as a critical component in the allocation of physician responsibilities for this objective (9).

Global collaborative research is crucial for improving our understanding of critical pediatric care illnesses such as sepsis (12) and pediatric acute respiratory distress syndrome (13), in hospitals of all resource levels (14). The first phase in developing strategies to enhance outcomes and lessen inequities is the evaluation of approaches, resources, and results in centers with limited resources (15). The Institutional Review Board (IRB) is essential for protecting study participants and upholding ethical standards; however, challenges faced with regional IRBs or ethics committees have restricted the participation of certain sites in multicenter trials, thereby diminishing the advantages of such research for patients in those areas. Furthermore, language barriers may pose significant challenges to international research collaborations (9, 16).

This study emphasizes the significance of the differences in outcomes between participating and non-participating centers in a multinational low-risk study and suggests some strategies to try to overcome them. However, collaborative efforts are still required for the development of standardized guidelines and the comprehension of the relevance of these studies in improving outcomes. Establishing a more straightforward or uniform procedure for research involvement and addressing language barriers could be helpful in promoting increased participation from resource-limited centers. Moreover, centralized IRBs could expedite the approval procedure.

The strengths of this study include its focus on countries with limited resources, where exploring critical care conditions in children is especially valuable. These conditions may be influenced by resource availability, geographical location, and other contextual elements. This research possesses multiple limitations. Despite the Global PARITY being international, overrepresentation of centers in Latin America (7). Consequently, our findings regarding barriers to research may not comprehensively represent conditions in other resource-limited environments with distinct characteristics, such as language and temporal factors, necessitating further exploration of additional barriers in varied contexts. Additional research might explore into additional challenges and potential solutions in different sorts of studies and settings.

## 5 Conclusion

Global collaborative research is essential, and IRBs are critical to ensure that this research complies with ethical standards, but the benefits of this kind of research may be constrained by obstacles to IRB approval. Critical barriers to study site participation were absence of institutional support for research, which coexisted with staffing shortages, restricted protected research time, financial assistance, and inadequate training, which are modifiable factors. These barriers restrict research activities in some resource limiting countries. Increasing the efficiency and accessibility of local IRB review could greatly impact participation of resource limited sites and enrollment of vulnerable populations.

## Data Availability

The original contributions presented in the study are included in the article/Supplementary Material, further inquiries can be directed to the corresponding author, Kenneth Remy, kenneth.remy@uhhospitals.org.
